# Effect of a postpartum family planning intervention on postpartum intrauterine device counseling and choice: evidence from a cluster-randomized trial in Tanzania

**DOI:** 10.1186/s12905-020-00956-0

**Published:** 2020-05-12

**Authors:** Erin Pearson, Leigh Senderowicz, Elina Pradhan, Joel Francis, Projestine Muganyizi, Iqbal Shah, David Canning, Mahesh Karra, Nzovu Ulenga, Till Bärnighausen

**Affiliations:** 1 Ipas, Chapel Hill, NC USA; 2 University of Wisconsin – Madison School of Medicine and Public Health, Madison, WI USA; 3 World Bank, Washington, DC USA; 4 Department of Family Medicine and Primary Care, School of Clinical Medicine, University of the Witwatersrand, Johannesburg, South Africa; 5 Association of Gynaecologists and Obstetricians of Tanzania (AGOTA), Dar es Salaam, Tanzania; 6 Muhimbili University of Health and Allied Sciences, Dar es Salaam, Tanzania; 7 Harvard T.H. Chan School of Public Health, Boston, MA USA; 8 Boston University, Boston, MA USA; 9 Management and Development for Health, Dar es Salaam, Tanzania; 10 Heidelberg Institute of Global Health, University of Heidelberg, Heidelberg, Germany

**Keywords:** PPIUD, Counseling, Tanzania

## Abstract

**Background:**

The World Health Organization recommends postpartum family planning (PPFP) for healthy birth spacing. This study is an evaluation of an intervention that sought to improve women’s access to PPFP in Tanzania. The intervention included counseling on PPFP during antenatal and delivery care and introducing postpartum intrauterine device (PPIUD) insertion as an integrated part of delivery services for women electing PPIUD in the immediate postpartum period.

**Methods:**

This cluster-randomized controlled trial recruited 15,264 postpartum Tanzanian women aged 18 or older who delivered in one of five study hospitals between January and September 2016. We present the effectiveness of the intervention using a difference-in-differences approach to compare outcomes, receipt of PPIUD counseling and choice of PPIUD after delivery, between the pre- and post-intervention period in the treatment and control group. We also present an intervention adherence-adjusted analysis using an instrumental variables estimation.

**Results:**

We estimate linear probability models to obtain effect sizes in percentage points (pp). The intervention increased PPIUD counseling by 19.8 pp (95% CI: 9.1 – 22.6 pp) and choice of PPIUD by 6.3 pp (95% CI: 2.3 – 8.0 pp). The adherence-adjusted estimates demonstrate that if all women had been counseled, we would have observed a 31.6 pp increase in choice of PPIUD (95% CI: 24.3 – 35.8 pp). Among women counseled, determinants of choosing PPIUD included receiving an informational leaflet during counseling and being counseled after admission for delivery services.

**Conclusions:**

The intervention modestly increased the rate of PPIUD counseling and choice of PPIUD, primarily due to low coverage of PPIUD counseling among women delivering in study facilities. With universal PPIUD counseling, large increases in choice of PPIUD would have been observed. Giving women informational materials on PPIUD and counseling after admission for delivery are likely to increase the proportion of women choosing PPIUD.

**Trial registration:**

Registered with clinicaltrials.gov (NCT02718222) on March 24, 2016, retrospectively registered.

## Background

The World Health Organization (WHO) recommends postpartum family planning (PPFP) for healthy birth spacing [1]. PPFP is defined as FP use within the first year postpartum when subsequent pregnancies are risky for maternal and child health outcomes [2]. Fertility can return as soon as 45 days after giving birth for women who are not breastfeeding [3], and among women who are not breastfeeding exclusively, fertility can return before the resumption of menses [1]. Provision of PPFP immediately following delivery may be appealing for women who prefer to ensure postpartum protection as the timing of fertility return may be difficult to predict, and WHO recommends that all women are offered a method within 6 weeks postpartum [4]. However, unmet need for family planning is high in the postpartum period, ranging from 32 to 62% in low and middle-income countries depending on the definition used [5].

WHO recommends lactational amenorrhea (LAM), condoms, male or female sterilization, progesterone-only pills, implants, and the copper intrauterine device (IUD) immediately following delivery for women who plan to breastfeed [6]. Other methods, including injectables, combined hormonal contraceptive pills and emergency contraception can be used by non-breastfeeding women [6]. Postpartum IUD (PPIUD) insertion has been lauded as a good option for those who lack regular access to health services because it can be inserted immediately following delivery before the woman is discharged from the health facility. However, PPIUD insertion requires specialized skills and is often unavailable in health facilities that offer delivery services in low- and middle-income countries [7].

In Tanzania, the median inter-birth interval has increased over time and most recent estimates report an inter-birth interval of 35 months [8], which is in line with WHO recommendations, but evidence suggests that some subgroups of women such as young women and those with lower educational status are more likely to have short inter-birth intervals [9]. Postpartum family planning use is low in Tanzania with 23% of women using a method of family planning by 6 months postpartum and 30% by 12 months postpartum [10]. Method mix in the postpartum period mirrors that in the general population in Tanzania with the exception of higher rates of LAM in the postpartum group (25.9%). Other commonly used methods during the postpartum period include injectables (22.5%) and pills (13.5%). PPFP use varies by sociodemographic characteristics with urban, wealthier, more educated women using PPFP at significantly higher rates [10]. Women with higher numbers of antenatal care visits and those having facility-based deliveries compared to home deliveries had only slightly higher rates of PPFP use in Tanzania, suggesting room for improvement in postpartum family planning programs.

The present study is an evaluation of an intervention that sought to improve women’s access to PPFP in large, tertiary care facilities in Tanzania. The intervention focused on increasing counseling on PPFP during antenatal care (ANC) visits and integrating PPIUD insertion within delivery services for women choosing PPIUD in the immediate postpartum period. The analysis focuses on the effect of the intervention on this newly added service, including effects on PPIUD counseling and women’s choice of PPIUD (i.e. having a PPIUD inserted) before being discharged from the hospital after delivery. We also assess factors associated with choice of PPIUD, including measures of counseling quality and women’s socio-demographic characteristics. The intervention was implemented by the International Federation of Obstetricians and Gynecologists (FIGO) in partnership with its Tanzanian affiliate, the Association of Gynecologists and Obstetricians of Tanzania (AGOTA), as part of a larger project that implemented and evaluated the FIGO PPFP intervention in three countries: Tanzania, Nepal and Sri Lanka. The results of the evaluations in Nepal and Sri Lanka are published elsewhere [11, 12].

## Methods

Data were collected through a cluster-randomized stepped-wedge trial to evaluate the impact of an intervention that introduced PPIUD services in six tertiary health facilities in Tanzania. The trial was registered with clinicaltrials.gov (NCT02718222), and the full study protocol has been published elsewhere [13]. The study also received ethical approval from the National Institute of Medical Research (NIMR) in Tanzania (protocol number: NIMR/HQ/R.8a/Vol.IX/2006). The study received a human subjects exemption from the institutional review board at Harvard University (protocol number IRB15–1605) as only de-identified data were received by the Harvard evaluation team.

### Study design and deviations from the stepped-wedge protocol

For this cluster-randomized stepped-wedge trial, six large, tertiary care facilities were selected by AGOTA, the implementing agency for the intervention, to provide coverage of PPIUD services for different regions of Tanzania. The stepped-wedge design was selected to measure intervention effectiveness because it is characterized by staggered intervention implementation in all study facilities, which ensured that all women delivering in study facilities could potentially benefit from the intervention. The evaluation team matched facilities in pairs based on annual delivery caseload, and within each pair, one facility was randomly assigned to Group 1 (early intervention implementation) and the other to Group 2 (late intervention implementation). The matched pair group assignments were as follows: Dodoma General Hospital in Dodoma (Group 1) and Mt. Meru Hospital in Arusha (Group 2), Muhimbili National Hospital in Dar es Salaam (Group 1) and Sekou-Toure Regional Referral Hospital in Mwanza (Group 2), and Mbeya Zonal Referral Hospital in Mbeya (Group 1) and Tumbi-Piwani Regional Referral Hospital in Kibaha (Group 2).

After randomization, there were two key deviations from the stepped-wedge protocol. First, before data collection started, the evaluation team decided to drop Sekou-Toure Regional Referral Hospital from the evaluation because the hospital served as a family planning model facility for the country and had an ongoing PPIUD intervention, which would make it difficult to isolate the effect of the newly implemented FIGO/AGOTA intervention. Data for the evaluation were only collected in the remaining five hospitals. Second, significant delays in intervention implementation in the Group 2 hospitals (Tumbi-Pwani Regional Referral Hospital and Mt. Meru Hospital) led to insufficient data collected after intervention implementation began. Group 2 hospitals were scheduled to start intervention implementation on 15th September 2016, but implementation was delayed until 17th November 2016, 1 month before the end of data collection, providing data for only 1 month rather than the planned 3 months. As a result, we have dropped the intervention period for the Group 2 hospitals and will consider the Group 2 hospitals as control facilities that are only observed in a state where they do not receive the intervention even as the Group 1 hospitals receive the intervention. This set-up of the data allows us to conduct the analysis as a treatment/control study of a cluster-randomized trial using a difference-in-difference approach. The difference-in-difference approach compares the change in an outcome that is observed in the Group 1 (treatment) hospitals between the pre- and post-intervention periods relative to the change in the outcome that is observed in the Group 2 (control) hospitals over the same period of time. The key identifying assumption of this analytic approach, referred to as the “parallel trends” assumption, is that the change in the outcome in the treatment hospitals between the pre- and post- periods would have been the same as the observed change in the control hospitals over the same period had the treatment hospitals not received the intervention. More specifically, the average outcome in the two groups would have evolved in parallel over time in the absence of the intervention, even if the average outcome between the Group 1 hospitals and Group 2 hospitals in the period had differed in the pre-period, before the Group 1 hospitals received the intervention. In this manner, any deviation from the relative parallel trend of the outcome into the post-intervention period between the Group 1 and Group 2 hospitals can be attributed to the effect of intervention on the outcome. On 15th January 2016, data collection commenced in both Group 1 and Group 2 hospitals, and the analysis will consider only the initial 8 months of data collection (15th January 2016 – 15th September 2016), before intervention implementation was to take place in the Group 2 hospitals. Group 1 hospitals began implementing the intervention in mid-May 2016, providing 4 months of data during the pre-intervention period and 4 months of data during the post-intervention period.

### Intervention

The intervention sought to improve women’s access to PPFP through improved counseling during ANC and through the introduction of immediate PPIUD insertion services in health facilities. The intervention was implemented by FIGO in partnership with AGOTA. Specific intervention components included: 1) information education and communication (IEC) materials on PPFP, including leaflets and a video that played in the waiting room; 2) provider training on PPFP counseling and PPIUD insertion techniques; 3) provision of equipment, including Kelly’s forceps to insert the IUD; and 4) regular monitoring and support provided by FIGO and AGOTA. All four elements of the intervention were implemented in the three Group 1 hospitals, and counseling and IEC materials were also made available in satellite clinics surrounding the Group 1 hospitals where many women received ANC services before delivering in the study hospitals. The intervention was implemented in two stages: AGOTA first conducted a training of trainers (TOT) from each intervention hospital, and then trainers provided cascade training to Ob/Gyns, residents and midlevel providers in their hospital approximately 1 month later. The post-intervention period is considered to have started after the cascade training was completed in the Group 1 hospitals.

### Data collection

Trained Research Assistants with previous experience conducting surveys were posted in post-natal wards of study hospitals where they conducted an interviewer-administered survey with women who consented to participate. Research Assistants were employed by AGOTA to collect data over the full project implementation period, but they were managed by the local research organization, Management and Development for Health (MDH), during the evaluation data collection period. The women’s survey collected socio-demographic data, information on PPFP counseling, including timing of counseling and information about the birth and PPFP decision-making. In addition, providers completed a survey about PPIUD insertion for women choosing a PPIUD as their PPFP method before discharge from the hospital. All data were collected using pre-programmed tablets using the CommCare application by Dimagi.

### Outcomes of interest

The key outcomes of interest for this evaluation were counseling on PPIUD and choice of the PPIUD after delivery, as PPIUD insertion was a newly offered service after intervention implementation. Counseling on PPIUD was measured through women’s self-report, and a woman was considered to have been counseled if she reported PPIUD counseling during antenatal care or during her stay at the hospital for delivery. Choice of PPIUD was measured as a dichotomous variable based on both the woman’s report and the provider’s report of PPIUD insertion. Occasionally, a woman would choose to have a PPIUD inserted after she completed her survey, and the insertion would be reported only on the provider survey. If either the woman or the provider reported PPIUD insertion, the woman was considered to have chosen the PPIUD.

### Analytic sample

**Fig. 1:**
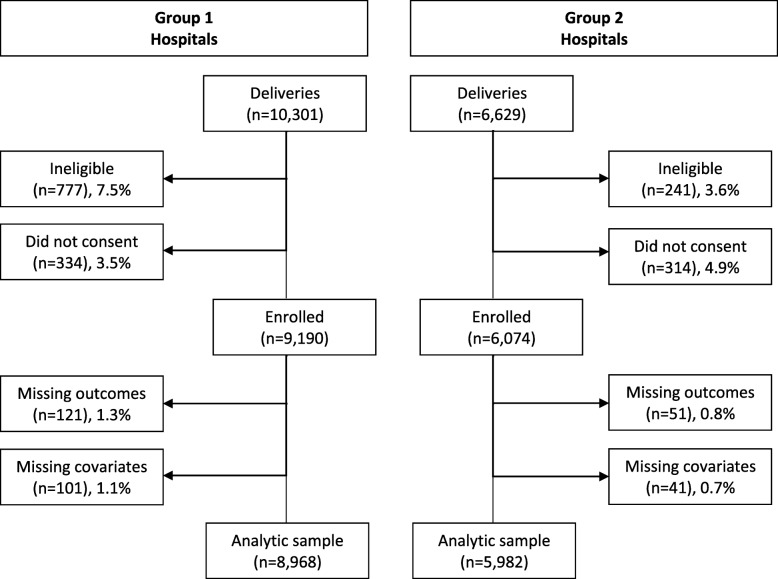
Study flow chart

A total of 16,930 women who delivered during the study period (15th January 2016 – 15th September 2016) in five hospitals were screened for study eligibility (age 18 or older, delivered in one of the five study hospitals, and resident of Tanzania), 15,912 (94%) were eligible (ineligibility primarily due to age under 18 years), and 15,264 (96%) of them consented to participate (Fig. 1). A total of 14,950 women with complete information on the outcomes of interest and key covariates were retained for the analysis (98% of those who consented to participate) – 8968 in Group 1 hospitals and 5982 in Group 2 hospitals. The CommCare data collection application required a response to each question, and missing data are due to participant refusal to give a response or ending the survey early.

### Analysis

We conducted a difference-in-difference analysis to evaluate the effect of intervention exposure, defined as delivering in an intervention hospital during the implementation period, on the two outcomes of interest: PPIUD counseling and choice of PPIUD. Linear probability models were used to estimate the effect of the intervention in percentage points (pp). In all models, we controlled for hospital and month fixed effects. We present an unadjusted model showing the effect of intervention exposure on each outcome controlling only for the hospital and month fixed effects and an adjusted model which includes women’s socio-demographic characteristics. Characteristics include woman’s age, education, parity, marital status, religion, and whether the woman was being seen in the “fast track” or normal track service. Fast track services cost more than normal track services and typically have better amenities and a lower provider to patient ratio.

Next, we measure the intervention adherence-adjusted effect of the intervention on choice of PPIUD. Some women were not exposed to the intervention for a variety of reasons, including inconsistent implementation of the intervention counseling, because they received ANC in a facility that did not offer counseling on PPIUD, or because they did not attend ANC services. The adherence-adjusted approach assumes that all of the effect of the intervention is through counseling and allows us to measure the effect of the intervention on choice of PPIUD among women who were counseled on PPIUD. A linear probability model is used to estimate the adherence-adjusted effect, which is equivalent to a standard instrumental variables (IV) approach [14].

We also present an analysis of the determinants of women’s choice of PPIUD among women who were counseled, controlling for hospital and month fixed effects. This analysis focuses on measured aspects of quality in counseling, including timing of counseling, whether IEC materials were used (leaflet given and video seen), whether they were given an opportunity to ask questions during counseling and the types of information they recall from the PPIUD counseling they received, and women’s socio-demographic characteristics that are associated with choice of PPIUD.

Due to the small number of clusters (hospitals) included in our analysis, all of our models adjust standard errors using the cluster wild bootstrapping method with Webb weights, a six-point distribution that reduces spurious precision due to replications based on a small number of clusters [15]. This approach produces corrected standard errors for all point estimates presented herein.

## Results

Table 1 presents the socio-demographic characteristics of women delivering in Group 1 and Group 2 hospitals. Most women were under age 30 years, had completed primary education, were currently married and had one child. Religion was evenly distributed across Catholic, Muslim, Lutheran/Anglican, and Pentecostal and Other Christian groups. Approximately 85% of women used normal track services. No statistically significant differences were observed by group. Table 2 presents characteristics of women delivering in the Group 1 and Group 2 hospitals during the pre-intervention period (mid-January – mid-May 2016). There was a low level of PPIUD counseling (~ 3%) and choice of PPIUD (0.7%) reported during the pre-intervention period. Socio-demographic and PPIUD characteristics did not vary between the two groups, demonstrating balance between Group 1 and Group 2 in the pre-intervention period.

**Table 1:** Background characteristics of women by group (*n* = 14,950)

	Group 1 percentage	Group 2 percentage	*p*-value
Woman’s Age			
18–20	11.8	8.4	0.229
20–24	30.2	32.8	0.508
25–29	27.5	29.0	0.680
30+	30.5	29.8	0.823
Woman’s Education			
Less than primary	6.0	4.3	0.487
Completed primary	53.4	51.1	0.811
Completed secondary	24.1	32.9	0.244
Some college	16.5	11.8	0.392
Parity			
1	46.9	43.2	0.411
2	21.7	25.1	0.330
3+	31.4	31.7	0.927
Marital Status			
Currently married/living with partner	93.6	92.8	0.671
Formerly married/widowed	0.4	0.6	0.205
Never married/never lived together	6.0	6.6	0.726
Religion			
Catholic	19.7	23.9	0.095
Muslim	21.5	29.4	0.626
Lutheran and Anglican	27.5	32.4	0.775
Pentecostal and Other Christian	31.3	14.3	0.525
Hospital Track			
Normal track	84.9	85.5	0.966
Fast track	15.1	14.5	0.966
**Observations**	8968	5982	

Note: *p*-values calculated using Wild Cluster Boostrap method

**Table 2:** Background characteristics of women by group during the pre-intervention period (*n* = 7145)

	Group 1 Pre-Intervention percentage	Group 2 Pre-Intervention percentage	Difference [Group 2 percentage - Group 1 percentage]	*p*-value
Woman’s Age (years)				
18–20	12.3	8.1	−4.1	0.183
20–24	29.9	33.5	3.7	0.489
25–29	27.6	30.0	2.4	0.556
30+	30.3	28.3	−1.9	0.700
Woman’s Education				
Less than primary	7.0	4.4	−2.6	0.345
Completed primary	50.8	51.0	0.2	0.961
Completed secondary	24.2	33.3	9.1	0.127
Some college	18.0	11.3	−6.8	0.293
Parity				
1	46.8	43.2	−3.6	0.400
2	22.3	25.5	3.2	0.313
3+	30.9	31.3	0.4	0.901
Marital Status				
Currently married/living with partner	92.3	93.2	0.9	0.513
Formerly married/widowed	0.5	0.7	0.1	0.550
Never married/never lived together	7.2	6.2	−1.0	0.485
Religion				
Catholic	22.8	24.4	1.7	0.554
Muslim	21.6	29.0	7.4	0.623
Lutheran and Anglican	26.7	31.6	4.9	0.749
Pentecostal and Other Christian	29.0	15.1	−13.9	0.554
Hospital Track				
Normal track	80.3	79.8	−0.5	0.974
Fast track	19.7	20.2	0.5	0.974
Received PPIUD Counseling	3.7	3.5	−0.2	0.648
Chose PPIUD	0.7	0.4	− 0.3	0.374
**Observations**	4224	2921		

Note: *p*-values calculated using Wild Cluster Boostrap method

**Fig. 2:**
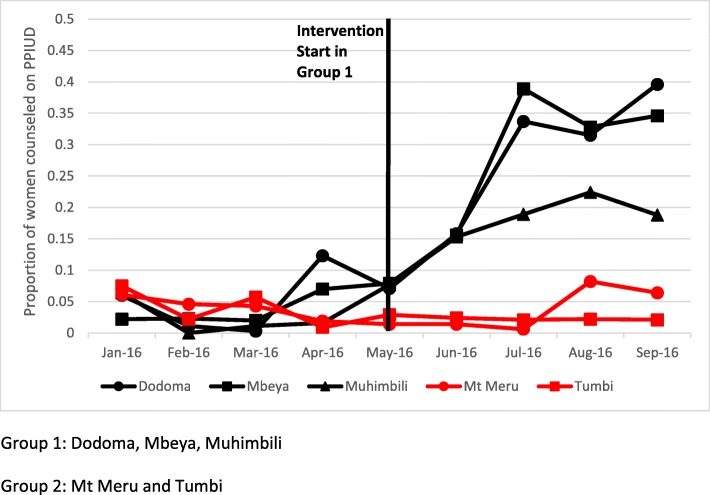
Proportion of women counseled on PPIUD, by group and hospital (*n* = 14,950)

**Fig. 3:**
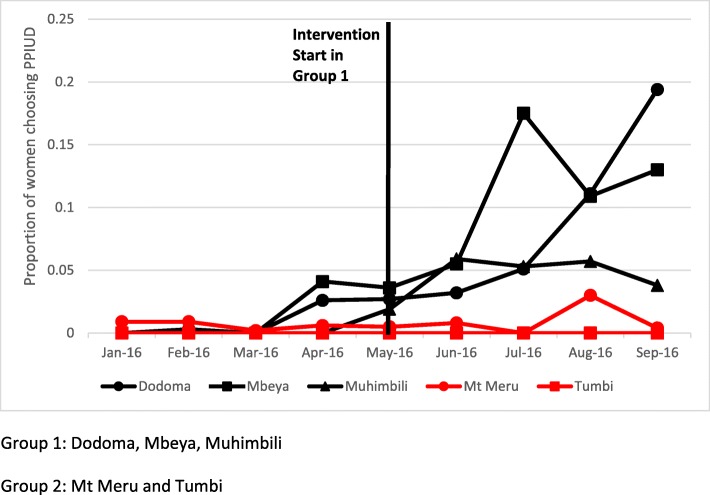
Proportion of women choosing PPIUD, by group and hospital (*n* = 14,950)

Figures 2 and 3 present trends in the two outcomes of interest, PPIUD counseling and choice of PPIUD, over the data collection period. During the pre-intervention period, PPIUD counseling and insertion rates were low in Group 1 (black line) and Group 2 (red line). The small uptick in PPIUD counseling and insertion in Group 1 hospitals in April 2016 (1 month before intervention implementation officially began) is due to the TOT that occurred in the Group 1 hospitals during this month. The trainers first received classroom training then after sufficient practice, they continued their training with live clients. As a result, trainers counseled some women on PPIUD and provided PPIUD during this period in April 2016, but we do not consider the intervention to have started until the full cascade training took place in mid-May 2016. After the intervention began in mid-May 2016, counseling rates steadily increased in Group 1 hospitals up to approximately 40% in Dodoma and Mbeya Hospitals and up to 22% in Muhimbili National Hospital, while counseling rates in Group 2 hospitals remained low as they received no intervention during the study period (Fig. 2). Overall, 24% of women were counseled on PPIUD in Group 1 hospitals during the post-intervention period. A similar pattern is observed in choice of PPIUD with insertion rates increasing in Group 1 hospitals after the start of intervention implementation (Fig. 3). Women’s choice of PPIUD varied between Group 1 hospitals with a maximum of approximately 20% selecting PPIUD in Dodoma, 18% in Mbeya, and 6% in Muhimbili National Hospital in a given month over the four-month post-intervention period. Overall, 8% of women chose a PPIUD in Group 1 hospitals during the post-intervention period.

Table 3 presents PPIUD counseling characteristics and women’s knowledge of PPIUD among those who were counseled in Group 1 hospitals during the post-intervention period. Timing of PPIUD counseling varied with 32.0% being counseled only during ANC, 43.0% being counseled only after admission for delivery, and 25.0% being counseled at both times. Exposure to the IEC materials was low among those who were counseled with only 10.7% reporting that they received the PPFP leaflet during counseling and only 10.3% reporting that they saw the PPFP video in the waiting room. Fewer than half of women (41.0%) reported being given an opportunity to ask questions during counseling. Most women reported that they recalled being counseled only on benefits of PPIUD (62.7%), and only 18.9% recalled both benefits and disadvantages of the method.

**Table 3:** Characteristics of counseling on PPIUD and PPIUD knowledge among women counseled on PPIUD during the post-intervention period in Group 1 hospitals (*n* = 1214)

	n	%
Timing of PPIUD Counseling		
Before admission/during ANC	390	32.0
After admission	525	43.0
Both	305	25.0
Given Leaflet during Counseling	131	10.7
Saw Video at Health Facility	126	10.3
Given Opportunity to Ask Questions	498	41.0
Knowledge about PPIUD		
Cannot recall any information or recall only disadvantages	224	18.4
Recall only benefits	765	62.7
Recall both benefits and disadvantages	231	18.9
**Observations**	1214	

### Difference-in-difference analysis

Table 4 presents the difference-in-difference analysis results for PPIUD counseling. The effect of the intervention was an increase of 19.8 pp in PPIUD counseling (95% CI: 9.1 – 22.6 pp) between the pre- and post-intervention periods in Group 1 (treatment) compared to Group 2 (control). We found that PPIUD counseling varied across some subgroups of women. Women with three or more children were more likely to be counseled on PPIUD than women with one child, and Muslim and Lutheran/Anglican women were less likely to be counseled on PPIUD compared to Catholic women.

**Table 4:** Effect of intervention on receipt of PPIUD counseling (*n* = 14,950)

	Est.		95% CI	Est.		95% CI
Post-intervention (Ref: Pre-intervention)	0.195	*	[0.085–0.245]	0.198	*	[0.091–0.226]
Woman’s Age (Ref: 18–20)						
20–24				−0.011		[− 0.035–0.017]
25–29				0.003		[− 0.014–0.022]
30+				0.009		[−0.011–0.022]
Woman’s Education (Ref: Less than primary)						
Completed primary				0.001		[−0.079–0.072]
Completed secondary				0.038		[−0.013–0.103]
Some college				0.041		[−0.043–0.111]
Parity (Ref: 1)						
2				0.030		[− 0.001–0.051]
3+				0.049	*	[0.011–0.082]
Marital Status (Ref: Currently married/living with partner)						
Formerly married/widowed				0.030		[−0.09–0.197]
Never married/never lived together				−0.000		[−0.014–0.025]
Religion (Ref: Catholic)						
Muslim				−0.021	*	[−0.045 - -0.005]
Lutheran and Anglican				−0.036	*	[−0.07 - -0.002]
Pentecostal and Other Christian				−0.033		[−0.065–0.011]
Fast Track (Ref: Normal track)				0.013		[−0.031–0.153]
Constant	0.056	*	[0.002–0.133]	0.044		[− 0.072–0.196]
**Observations**	14,950			14,950		
**R-squared**	0.122			0.133		

**p < 0.01, *p < 0.05,

Note: *p*-values calculated using Wild Cluster Boostrap method. Regression models include month and hospital fixed effects

Table 5 presents the difference-in-difference analysis results for choice of PPIUD after delivery. The effect of the intervention was an increase of 6.3 pp in choice of PPIUD (95% CI: 2.3 – 8.0 pp) between the pre- and post-intervention periods in Group 1 (treatment) compared to Group 2 (control). Choice of PPIUD varied only by religion. Muslim women were less likely to choose PPIUD compared to Catholic women.

**Table 5:** Effect of intervention on women’s choice of PPIUD (n = 14,950)

	Est.		95% CI	Est.		95% CI
Post-intervention (Ref: Pre-intervention)	0.062	*	[0.021–0.081]	0.063	*	[0.023–0.08]
Woman’s Age (Ref: 18–20)						
20–24				−0.008		[−0.021–0.008]
25–29				−0.002		[−0.016–0.019]
30+				−0.001		[−0.008–0.01]
Woman’s Education (Ref: Less than primary)						
Completed primary				−0.015		[−0.054–0.022]
Completed secondary				−0.017		[−0.053–0.007]
Some college				−0.020		[−0.054–0.007]
Parity (Ref: 1)						
2				0.010		[− 0.001–0.024]
3+				0.025		[−0.003–0.056]
Marital Status (Ref: Currently married/living with partner)						
Formerly married/widowed				−0.003		[−0.028–0.05]
Never married/never lived together				−0.004		[−0.015–0.01]
Religion (Ref: Catholic)						
Muslim				−0.013	*	[−0.04 - -0.002]
Lutheran and Anglican				−0.008		[−0.027–0.005]
Pentecostal and Other Christian				−0.015		[−0.025–0.002]
Fast Track (Ref: Normal track)				0.003		[−0.012–0.025]
Constant	0.000		[−0.029–0.035]	0.014		[−0.036–0.06]
**Observations**	14,950			14,950		
**R-squared**	0.051			0.059		

**p < 0.01, *p < 0.05

Note: *p*-values calculated using Wild Cluster Boostrap method. Regression models include month and hospital fixed effects

### Analysis Adjusting for Intervention Adherence

Due to the relatively low rates of PPIUD counseling during the post-intervention period, we sought to adjust the effect size estimate for intervention adherence, i.e., whether a woman was counseled on choice of PPIUD after delivery. We counted both counseling during an ANC visit or at the hospital during delivery care as adherent to the intervention. A direct estimate of counseling on choice of PPIUD is likely to be biased. Table 3 shows that counseling varied across women’s socio-demographic characteristics, suggesting targeted rather than universal counseling. The adherence-adjusted approach assumes that all of the effect of the intervention is through counseling and allows us to estimate the effect of counseling on choice of PPIUD if all women had been counseled. Table 6 presents the adherence-adjusted results, and estimates suggest a 31.6 pp (95% CI: 24.3 – 35.8 pp) increase in choice of PPIUD if all women had been counseled.

**Table 6:** Adherence-adjusted effect of PPIUD counseling on choice of PPIUD (*n* = 14,950)

	Est.		95% CI	Est.		95% CI
Counseled on PPIUD (Ref: Not Counseled)	0.318	*	[0.255–0.351]	0.316	*	[0.243–0.358]
Woman’s Age (Ref: 18–20)						
20–24				−0.004		[−0.02–0.008]
25–29				−0.003		[−0.018–0.024]
30+				−0.003		[−0.011–0.006]
Woman’s Education (Ref: Less than primary)						
Completed primary				−0.016	*	[− 0.036 - -0.032]
Completed secondary				−0.029	*	[−0.049 - -0.009]
Some college				−0.033	*	[−0.05 - -0.011]
Parity (Ref: 1)						
2				0.001		[− 0.007–0.007]
3+				0.009		[−0.011–0.03]
Marital Status (Ref: Currently married/living with partner)						
Formerly married/widowed				−0.013		[−0.024–0.008]
Never married/never lived together				−0.004		[−0.013–0.002]
Religion (Ref: Catholic)						
Muslim				−0.007		[−0.033–0.005]
Lutheran and Anglican				0.003		[−0.004–0.01]
Pentecostal and Other Christian				−0.004		[−0.017–0.002]
Fast Track (Ref: Normal track)				−0.001		[−0.015–0.005]
Constant	− 0.018	*	[− 0.032 - -0.009]	0.000		[−0.016–0.013]
**Observations**	14,950			14,950		
**R-squared**	0.214			0.219		

**p < 0.01, *p < 0.05

Note: *p*-values calculated using Wild Cluster Boostrap method. Regression models include month and hospital fixed effects

### Determinants of choosing PPIUD

Table 7 presents the determinants of choice of PPIUD among women who were counseled. Women who were counseled after admission were more likely to choose PPIUD compared to women who were only counseled before admission/during ANC. Receipt of the PPFP leaflet was also associated with choice of PPIUD. Among those who were counseled, Muslim women were less likely to choose the PPIUD compared to Catholic women.

**Table 7:** Determinants of women’s choice of PPIUD among women who were counseled (n = 1214)

	Est.		95% CI
Timing of PPIUD Counseling (Ref: Before admission/during ANC)			
After admission	0.2467	*	[0.069–0.403]
Both	0.2970		[−0.437–1.005]
Leaflet given during counseling (Ref: No leaflet given)	0.4095	*	[0.113–0.846]
Saw video at health facility (Ref: Did not see video)	0.0642		[−0.549–0.322]
Given Opportunity to Ask Questions (Ref: No opportunity given)	0.1633		[−0.906–1.239]
Knowledge about PPIUD (Ref: Cannot recall or recall only disadvantages)			
Recall only benefits	−0.0338		[−0.639–0.543]
Recall both benefits and disadvantages	0.1980		[−0.324–0.815]
Woman’s Age (Ref: 18–20)			
20–24	−0.0056		[−0.379–0.374]
25–29	0.0347		[− 0.498–0.723]
30+	0.0337		[− 0.477–0.699]
Woman’s Education (Ref: Less than primary)			
Completed primary	−0.1291		[−0.314–0.238]
Completed secondary	−0.2335		[−0.477–0.159]
Some college	−0.2208		[−0.433–0.058]
Parity (Ref: 1)			
2	−0.0426		[−0.474–0.445]
3+	0.0017		[−0.698–0.638]
Marital Status (Ref: Currently married/living with partner)			
Formerly married/widowed	0.1687		[−15.72–6.06]
Never married/never lived together	−0.0582		[−0.294–0.222]
Religion (Ref: Catholic)			
Muslim	−0.0391	*	[−0.147 - -0.026]
Lutheran and Anglican	0.0342		[−0.362–0.352]
Pentecostal and Other Christian	− 0.0482		[− 0.217–0.106]
Fast Track (Ref: Normal track)	−0.1063		[−0.378–0.145]
Constant	0.0819		[−1.543–1.606]
**Observations**	1214		
**R-squared**	0.3425		

**p < 0.01, *p < 0.05

Note: *p*-values calculated using Wild Cluster Boostrap method. Regression model includes month and hospital fixed effects

## Discussion

### Main findings

This study evaluates the effect of an intervention that sought to increase women’s access to PPIUD services immediately following delivery. We found that the intervention increased PPIUD counseling by 19.8 pp and choice of PPIUD by 6.3 pp. These increases are statistically significant but relatively modest, primarily due to low coverage of PPIUD counseling among women delivering in Group 1 (treatment) facilities during the post-intervention period. Adherence-adjusted estimates demonstrate that if all women had been counseled, we would have observed an increase of 31.6 pp in choice of PPIUD – a result five times higher than the observed increase.

### Strengths and limitations

The strength of this study is the randomized design. We achieved balance on the outcomes and important covariates between the two groups during the pre-intervention period, suggesting that any differences in outcomes during the post-intervention period can be attributed to the intervention. One limitation is that intervention implementation took place only in tertiary care facilities, and findings may not generalize to similar interventions that are implemented in lower level health facilities.

### Interpretation

Intervention implementation varied across the Group 1 hospitals with Dodoma and Mbeya performing better than Muhimbili National Hospital. Muhimbili National Hospital is the national referral hospital in Tanzania and sees some of the most complicated cases. For this reason, a service such as PPIUD may be a low priority compared to other life-saving treatments needed in this complicated patient population. However, even in Dodoma and Mbeya hospitals, fewer than 40% of delivery clients reported being counseled on PPIUD. Low rates of counseling may be due to inconsistent implementation or women seeking ANC from facilities where the intervention was not being implemented. Since study hospitals were referral facilities, many of the women delivering may have been referred from distant facilities and may not have had an opportunity to be counseled during ANC at the hospital or its surrounding satellite clinics where intervention implementation occurred. However, all women delivering during the post-intervention period in Group 1 hospitals had an opportunity to be counseled after admission for delivery.

Among women who were counseled on PPIUD, 57.0% reported counseling during ANC or both during ANC and after admission for delivery, and the remaining 43.0% only received counseling after admission for delivery. The intervention initially sought to counsel only during ANC to maximize the amount of time women had for informed decision-making, but counseling was also offered at the time of delivery to ensure that any women who wanted to use the PPIUD had the opportunity. The adjusted model found that women who were counseled after admission were more likely to choose PPIUD, corroborating findings from a recent review article, which found that interventions providing counseling in postnatal wards are effective in increasing postpartum contraceptive uptake [16].

Quality of counseling was relatively low among women who were counseled on PPIUD. Very few women who were counseled received the PPFP leaflet or saw the video. The adjusted model found that receiving the leaflet was associated with choice of PPIUD, which suggests that women who had time to review the information and possibly to share it with a husband/partner or other family members or friends were more likely to choose PPIUD than those who did not have this opportunity. Other studies have also identified leaflets provided during ANC as important for increasing uptake of PPIUD [17]. We also found that quality of counseling was relatively low with fewer than half of women given an opportunity to ask questions during counseling, and only 18.9% recalling balanced information on PPIUD, including its benefits and disadvantages. Though knowledge about PPIUD was not associated with choice of PPIUD in the adjusted model in this study, receipt of balanced counseling is a goal for high quality family planning programs and knowledge of PPIUD has been shown to be associated with higher rates of PPIUD uptake in Sri Lanka and Nepal [11, 12].

## Conclusions

The intervention increased the rate of PPIUD counseling and choice of PPIUD. However, counseling rates were relatively modest, and counseling was not universally provided, with higher parity women and women from some religious groups more likely to receive counseling. We estimate that if universal counseling had been provided, five times more women would have chosen PPIUD. We also find that provision of IEC materials and counseling after admission for delivery are associated with choice of PPIUD. Improving coverage and quality of counseling is likely to increase women’s access to PPFP services, including PPIUD.

## Data Availability

Data and materials from the study may be available upon request from Nzovu Ulenga at Management and Development for Health (MDH), nulenga@mdh.or.tz or P.O. Box 79810, Plot #802, Mwai Kibaki Road, Mikocheni, Dar es Salaam, Tanzania.

## Abbreviations

AGOTA: Association of Gynecologists and Obstetricians of Tanzania
ANC: Antenatal care
FIGO: International Federation of Gynaecology and Obstetrics
IEC: Information education and communication
IUD: Intrauterine device
IV: Instrumental variables
LAM: Lactational amenorrhea
MOHCDE: Ministry of Health, Community Development, Gender, Elderly and Children
PPFP: Postpartum family planning
PPIUD: Postpartum intrauterine device
TOT: Training of trainers
WHO: World Health Organization
